# The Gut Microbiome May Help Address Mental Health Disparities in Hispanics: A Narrative Review

**DOI:** 10.3390/microorganisms10040763

**Published:** 2022-03-31

**Authors:** Fernando Vera-Urbina, María F. Dos Santos-Torres, Filipa Godoy-Vitorino, Bianca A. Torres-Hernández

**Affiliations:** 1 Faculty of Natural Sciences, University of Puerto Rico at Rio Piedras, San Juan 00925, Puerto Rico; fernando.vera1@upr.edu (F.V.U.); maria.dossantos@upr.edu (M.F.D.S.T.); 2 Department of Microbiology, School of Medicine, Medical Sciences Campus, University of Puerto Rico, San Juan 00925, Puerto Rico; filipa.godoy@upr.edu; 3 Department of Pharmaceutical Sciences, School of Pharmacy, Medical Sciences Campus, University of Puerto Rico, P.O. Box 365067, San Juan 00936, Puerto Rico

**Keywords:** gut–brain axis, microbiome, Hispanic, anxiety, depression

## Abstract

The gut–brain axis is the biological connection between the enteric and the central nervous systems. Given the expansion of the microbial sciences with the new human microbiome field facilitated by the decrease in sequencing costs, we now know more about the role of gut microbiota in human health. In this short review, particular focus is given to the gut–brain axis and its role in psychiatric diseases such as anxiety and depression. Additionally, factors that contribute to changes in the gut–brain axis, including the gut microbiome, nutrition, the host’s genome, and ethnic difference, are highlighted. Emphasis is given to the lack of studies on Hispanic populations, despite the fact this ethnic group has a higher prevalence of anxiety and depression in the US.

## 1. Introduction

Our gut feels different when we are nervous, sad, or have a busy day. The gut–brain axis or brain-gut axis (sometimes used interchangeably) is the biological link between the physiology and these emotions. It is described as a bidirectional communication system between the central nervous system (the brain and spinal cord) and the enteric nervous system [1]. In other words, it is the link between the brain’s emotional and rational parts and our intestines’ physiology and metabolism. Abnormal gut–brain axis activity has shown an association with physical and psychological illnesses. In the last decade, seminal work has demonstrated the importance of the gut microbiome and its influence on the system’s functionality [1,2,3,4,5]. 

The collective genome content of the microbiota, the collection of microbial communities inhabiting the human body, was coined the microbiome [6]. The host–microbiome supraorganism has co-evolved, shaping the physiological phenotypes that lead to either health or disease [7,8,9]. The gut microbiota impacts diverse physiological processes ranging from metabolism [10,11], obesity [12,13], immune modulation [14], and even behavior [15,16,17,18]. In each case, the mechanistic ties of gut microbes and the host phenotypic responses are still unresolved, and much more research is needed. Ethnical changes associated with the microbiota are becoming more evident, suggesting that the microbiome may contribute to ethnic health disparities. Factors such as diet and lifestyle behaviors are the most important variables that influence health outcomes and simultaneously impact the human microbiome. Changes in these factors (i.e., nutrition and/or lifestyle) impact the microbiome and affect the host’s metabolic functions, which may underly all health inequities [19]. 

A multi-ethnic community study in Malaysia showed that ethnicity significantly impacted gut microbiota diversity. People from different ethnicities have different proportions of some types of bacteria due to the fact of diet, medication use, and hygiene [20]. Similarly, significant differences in gut microbiota have been found among peoples of different ethnicities in the US. The gut microbiota of Caucasians compared to Asian, Pacific Islanders, and Hispanics have significant differences at the family level including in *Christensenellacea* distinguishing Asian–Pacific islanders [21]. Another study conducted in Canada showed a higher abundance of lactic acid bacteria in children from South Asians when compared to Caucasians. The differences in bacterial abundance were associated with ethnicity and breastfeeding. These two main factors independently influenced infant gut microbiome at one year of age [22]. 

In addition, psychological illnesses are linked with altered intestinal functions, microbiota composition, and diversity [23]. In 2019, among systematic reviews, there were fewer than ten investigations that explored the connection between major depressive disorder (MDD) or bipolar disorder with human gut microbiota [4,24]. Some reported a significant difference in bacterial diversity in MDD patients versus control groups, while others failed to find any difference.

Anxiety and depression are currently the most common psychological illnesses globally. In the US, the prevalence increased during the first months of the COVID-19 pandemic. Since March 2020, the prevalence of anxiety and/or depression has almost tripled compared to 2019; it increased from 11% to 33.9% by May 2020 [25]. At the beginning of the lockdown in the US, Hispanics/Latinos on the mainland had a higher prevalence of psychosocial stress related to food insecurity than other ethnic/racial group in the US [26]. 

Gut microbiota changes have thus been linked to health inequality [19]. Nonetheless, the results have varied, and some lead to contradictory results due to the different methodologies, sample collection methods, and data analyses, which means more investigations are needed. Clear guidelines are taught for standardized microbiome analyses. The data suggest that each ethnic group has unique genome expressions and gut microbiota compositions [27,28,29,30,31,32,33,34]. Several research programs seek to sequence the gut microbiome of different ethnic groups to learn more about the differences, but most of them have focused on samples from European or North Americans. Data from Hispanic populations, among other ethnic groups or races, are scarce [21,35]. In fact, this highlights the vast underrepresentation of microbiome research from low- and middle-income countries, which calls for a more sustainable and inclusive agenda for microbiome studies [36]. Unfortunately, most studies about the gut–brain axis have not explored the association in a particular ethnicity [4], leaving vast research opportunities. Indeed, we accessed PubMed on 14 January 2022 and found only 13 studies on gut microbiota in Hispanics related to behavior including stress; however, the majority were related to cardiovascular disease [37] and obesity [38]. This confirms a significant gap in gut microbiota studies linked to depression and other related disorders.

This review discusses supporting data about the association between the gut–brain-axis, gut microbiome, nutrition, and psychological illnesses. Lastly, we address why Hispanic populations are largely neglected and remain an essential focal point to be included in these multi-omic studies.

## 2. Revealing the Gut–Brain Axis

The enteric nervous system (ENS) is the gut’s autonomous outlying nervous system, also called the “second brain” [39]. It has two significant networks embedded in the digestive tract wall that extends from the esophagus to the anus. Both communicate and can influence each other [3]. ENS is a complex system with millions of neurons, glia cells, and interactions with different kinds of enteroendocrine cells [39]. Exchanges between these two systems include complex interactions involving nervous, endocrine, and immune signaling mechanisms [40]. Enteroendocrine cells are located in the bowel epithelium. They play an essential role in digestion. They produce hormones and neuropeptides, which regulate serotonin levels, gastric acid secretion, insulin secretion, appetite control, peristalsis, supporting vasodilatation, among other functions [39,41]. Research has indicated that there are possibly five communication routes between the brain and the gut: (1) the brain’s neural complex, (2) neuroendocrine–hypothalamic–pituitary–adrenal axis, (3) gut immune system, (4) neurotransmitters and neural regulators produced by gut bacteria, and (5) barrier pathways such as the blood–brain barrier [42]. In this review, we highlight the blood–brain barrier.

The blood–brain barrier (BBB) protects the brain by hindering most blood compounds from entering the brain [43]. Its primary purpose is brain homeostasis. Its compositions are capillary endothelial cells shut by tight junctions, astrocytes, and pericytes [44]. Dysfunction of the BBB results in increased cerebrovascular permeability, directly affecting neuronal activity [45]. It is known that the BBB integrity can be compromised as a response to gut microbiota metabolism. In an animal model, it has been observed that pathogen-free mice had greater BBB integrity when compared with their germ-free counterparts [44].

Additionally, conventionalized mice—which are germ-free adult mice inoculated with microbiota from pathogen-free mice–showed a lower BBB permeability than germ-free mice [44]. Researchers use specific pathogen-free mice under psychological stress fed regularly to evaluate the connection of the gut microbiota with the intestinal and BBB integrity. The results showed that stress led to weaker and lower expression of four tight junction proteins (i.e., claudin5, occludin, α-actin, and ZO-1) with broken membranes of the intestine barriers and BBB. Moreover, both groups showed differences in their microbiota composition [5]. Furthermore, the co-occurrence of psychiatric disorders and gastrointestinal conditions have been linked to perturbations of the gut–brain axis systems [1]. For example, irritable bowel syndrome (IBS) and inflammatory bowel disease (IBD) are typically presented with signs of psychiatric disorders such as anxiety, depression, somatization disorder, and/or bipolar disorder [46,47,48]. There is an increased interest in evaluating how the gut microbiota could impact health [49]. 

## 3. A Look into the Gut Microbiome

The microbiota refers to all microbial cells associated with living organisms and are mostly studies sequencing 16S rRNA genes identifying bacterial taxa, their relative abundance, and community structure. On the other hand, the microbiome includes the genetic information of the microbiota, its genes, and genomes—a term first coined by Handelsman [50]. Therefore, the microbiome is studied mainly through shotgun metagenomics, unveiling also the functional and gene capacity of the microbiota. As most studies do not clearly differentiate the terms microbiota and microbiome, and some consider 16S rRNA profiles as microbiome, in this article, the gut microbiota and microbiome will be used interchangeably. The “holobiont” consists of the host and its symbionts (prokaryotes and eukaryotes) a complex unit that has been transmitted during birth between generations through the matrilineal line since early human evolution [7,9]. The microbiome has many vital functions like producing energy and vitamins that are inaccessible to the host, helping to metabolize xenobiotics, preventing the colonization of different pathogens, and it is essential in developing the immune system [51,52]. The gut microbiome are all the microorganisms that reside in our intestines, and its gene makeup of the gut–brain axis has resulted in the term gut–microbiota–brain axis [1]. In the human microbiome, pathogenic and symbiotic bacteria, archaea, and fungi coexist in a healthy body. Dysbiosis occurs when that equilibrium is disrupted and beneficial bacteria disappear, which can be caused by infectious diseases, particular diets, or long-term use of antibiotics or other bacteria-killing drugs. Diseases, such as IBS, IBD, or colorectal cancer, can result from microbial dysbiosis induced by alterations in the microbial population composition, relative abundance, and metabolic profiles. As a result, the body’s susceptibility to disease increases. Multiple studies through the years have unveiled inter-individual differences of the gut microbiome [53], indicating that there is not one single definition of a healthy microbiome, highlighting that an appropriate anti-inflammatory fiber-rich diet and lifestyle are essential in maintaining the balance of gut probiotic taxa.

To understand this, most research has evaluated the gut microbiota using a limited population diversity, mostly in European and North American. Studies identifying the differential abundance of members of the microbiota by specific race or ethnic group could be useful [54,55].

The most abundant microorganisms in the human gut microbiome are *Bacteroidetes* and *Firmicutes* [56]. The *Firmicutes/Bacteroidetes* (F/B) ratio changes along with lifespan [57,58]. Six-week-old European infants evaluated for factors that could be influencing gut microbiota found that the most pronounced difference was geographical location. Dominant *bifidobacteria* was associated with Europe’s northern countries, while more diverse microbiota (with increased Bacteroides) was related to southern countries; they also evaluated delivery mode, breastfeeding, and the use of antibiotics as influencing factors [27]. *Actinobacteria*, *Bacteroidetes*, and *Firmicutes* phyla ratios are important in infants and children when studying their gut microbiota composition. An investigation on Asian newborns from different geographical locations (i.e., Singapore and Indonesia) found that *Bifidobacterium*, *Atopobium* (both belonging to *Actinobacteria* phylum), and Clostridium leptum (*Firmicutes* phylum) differed in abundance between the two groups [33]. Some researchers found a similar phyla composition of the gut microbiome across different populations when compared studies conducted in Korean, US, China, and Japanese populations [30]. However, a study performed in western Oklahoma found that Native American groups (i.e., Cheyenne and Arapaho) had a fecal metabolic profile related to metabolic disorders and lower levels of the genus *Faecalibacterium* (anti-inflammatory bacteria) compared to non-native people [31]. In addition, due to the multiple environmental conditions, Russian populations are an interesting population to evaluate gut microbiome. A descriptive analysis of healthy gut microbiomes showed the Russian gut composition was different from rural to urban people [32]. Moreover, their composition was mostly *Firmicutes* and *Actinobacteria* phylum. They had underrepresented Bacteroides-driven communities. They significantly differed in their gut microbiota compared with Chinese, Danish, and US samples [32].

The development of germ-free animal models was a great start for evaluating altered metabolomes. It has been beneficial for identifying primary metabolites produced by altered microbiota and also for developing association studies of the gut–brain axis. The short-chain fatty acids (SCFAs) were the first microbiome-derived metabolites to affect health. These are produced by the colonic fermentation of dietary fibers and absorbed by epithelial cells giving them energy [51]. Reduced levels of SCFAs are associated with inflammatory diseases such as IBD or Chron’s disease [59,60].

Germ-free (GF) mice (devoid of microbes and with an altered gut physiology), when compared to mice with a normal gut microbiota, revealed that products of the tryptophan metabolism associated with *Clostridium*, which are thought to affect neuronal signaling in the gut and brain, are absent in germ-free mice. Most research on depressive and stress behavior through the gut–brain axis also uses germ-free (GF) and specific pathogen-free (SPF). To study the “mice’s stress levels”, the authors measured corticosterone levels (a hormone involved in regulating the metabolism in response to stress) and ACTH, which is an essential component in the hypothalamic–pituitary–adrenal axis. The results showed that GF mice had higher stress levels than SPF mice [61]. However, SPF and GF mice had lower cortisol levels after gut microbiota transplantation from control SPF mice. The results suggested a role of the gut microbiota in the regulation of the hypothalamus–pituitary–adrenal axis [62]. 

Other studies explored the association between depression and gut microbiota remodeling via fecal microbiota transplantation with fecal matter from MDD patients and healthy patients to germ-free mice. Germ-free mice receiving fecal microbiota transplants derived from MDD patients had depression-like behaviors compared with colonization with “healthy microbiota” from healthy controls [63]. 

In humans, fecal matter transplants (FMTs) have been used successfully to treat psychiatric patients in different situations. A study in China followed a woman with severe depression and unresponsive to pharmacological treatment, who received and FMT from a healthy donor and became symptom-free after six months [64]. Similar successful results were obtained in a man with depression, suffering from alopecia and daily diarrhea. After fecal transplantation, all the symptoms improved including alopecia [65]. However, more research is needed, including joint dietary strategies to maintain the balance of healthy gut microbiota. 

## 4. Interactions between Microbiota and Neurochemistry

Neurotransmitters play an important role in the communication between endothelial cells and the central nervous system. Glutamate is the main excitatory neurotransmitter in the brain, but also it is present in the gastrointestinal tract through dietary sources [66] and produced by bacteria such as *Lactobacillus plantarum*, *Bacteroides vulgatus*, and *Campylobacter jejuni* [67]. When secreted by bacteria, neuropod—enteroendocrine cells—known as neuropod cells, use glutamate as a neurotransmitter to transmit sensory signals to the brain through the vagus nerve [68]. 

On the other hand, γ-aminobutyric acid (GABA) is the main inhibitory neurotransmitter that gives sensory information to the brain through the vagus nerve and could decrease neurotransmitter levels associated with depression and anxiety [69]. GABA can be produced in the human gut by bacteria such as *Bifidobacterium*, *Bacteroides fragilis*, *Parabacteroides* [70], *Bacteroides caccae*, *Bacteroides vulgatus*, *Bacteroides ovatus*, *Bacteroides dorei*, *Bacteroides uniformis*, *Parabacteroides merdae*, *Eubacterium rectale*, and *Bifidobacterium adolescentis* [71]. GABA can be moved from the gut to the blood by transporters such as H^+^/GABA symport, found in the apical membrane of the intestinal epithelial cells passing through the gut epithelium [72]. However, there is still controversy over whether neurotransmitters can penetrate the blood–brain barrier. Studies have shown that neurotransmitters (or their precursor amino acids) could pass the BBB in association to stress factors [73]. Nevertheless, more research evidence is needed to determine the permeability of the BBB with neurotransmitters. Homotaurine is a BBB-permeable amino acid that antagonizes amyloid fibril formation, and it was found to ameliorate disease in mouse models of multiple sclerosis but had few benefits in long-term Alzheimer’s disease trials [74].

Bacteria, such as *Staphylococci*, can produce amino acid and neurotransmitters by biosynthesis process. *Staphylococci* in the human gut use phenylalanine, tyrosine, dihydroxy phenylalanine (L-DOPA), and 5-hydroxytryptophan (5-HTP) to produce phenylethylamine, tyramine, dopamine, and serotonin, respectively [75]. However, factors that influences gastric secretion, motility, and mucosal blood flow can alter the concentration of these molecules. Dopamine, which is associated with pleasure, cannot reach the brain through the blood–brain barrier [76,77], but dopamine passage from the gut to the brain and vice versa was found to be possible through the vagus nerve in mice [78]. 

Tryptophan is the precursor of serotonin, a neurotransmitter with an important role in depression. Humans can obtain it, either through diet or protein degradation [79]. Moreover, tryptophan can be produced by bacteria, such as *Lactococcus lactis* subsp. *cremori*, L. *lactis subsp. lactis*, *Lactobacillus plantarum*, *Streptococcus thermophilus*, *Escherichia coli*, *Morganella morganii*, and *Klebsiella pneumoniae*, and then be synthesized as serotonin in enterochromaffin cells in the gut [80]. Tryptophan can pass through the BBB [77]. Serotonin can send signals to the brain through enteric neurons and promote intestinal motility and secretions.

Other important pathways in the microbiota-gut–brain axis include the tryptophan-kynurenine metabolism. The microbiota can metabolize tryptophan to kynurenine, reducing the amount of tryptophan available [79]. An in silico study found that different gut phyla can produce other tryptophan metabolites, with Bacteroidetes, Actinobacteria, and Proteobacteria, producing more kynurenine [81]. A meta-analysis showed associated elevated levels of kynurenine to tryptophan in patients with psychiatric conditions such as depression and bipolar disorder. These data suggest that lower production of serotonin is not only due to the fact of a low pool of tryptophan but is also related to kynurenine conversion [82]. Supplementation with probiotics and prebiotics might decrease kynurenine concentration and the kynurenine:tryptophan ratio [83]; this suggests that probiotics and prebiotics could have clinical relevance as a treatment to regulate the tryptophan–kynurenine pathway [84]. Many other neurotransmitters have similar pathways enabling the communication between the brain and the gut microbiota. There is still much to be comprehended as for the relationship between neurotransmitters and the microbiota, leaving enormous opportunities for research in animal models, including non-human primates, which are phylogenetically, anatomically, and genetically close to humans.

## 5. The Importance of Nutrition

The food and respective nutrients that our body ingests are environmental factors that play a significant role in the gut microbiome’s diversity, composition, richness, and function [85]. Evaluating the diet and the gut microbiota in African and European children revealed a relevant difference in the gut composition. Data showed that rural African children had a higher amount of *Bacteroidetes* and lower *Firmicutes* than European children. In addition, rural African children showed an abundance of two genera, *Prevotella* and *Xylanibacter*, which were not present in European children [86]. Additionally, rural African populations are colonized by gut parasites, such as *Entamoeba*, as part of their gut microbiota [29].

Lastly, the most important shift seen in the gut microbiota has resulted from urbanization [87]. The migration of large numbers of people into cities as well as the Western lifestyle (fast food and lack of vegetables and fruits), has been associated with an increase in inflammatory diseases and decreased gut diversity [88,89,90]. Some studies suggest psychiatric disorders are also prevalent in these urban settings, providing a link to the gut microbiome axis [90,91].

A diet rich in different sources of fibers, fat, protein, and other micronutrients can also alter the gut microbiota. A diet low in nutrients tends to lead to the development of severe chronic conditions like diabetes, colorectal cancer, obesity, depression, and IBS [92]. High-fat diets can cause drastic and lasting alterations in the gut microbiota [93]. Association between the fatty acid intake and the abundance of *P. copri* was found in older Caribbean Latinos living in the US. The study also found that participants with type 2 diabetes had a greater abundance of *Enterobacteriales*, and those with obesity exhibited a higher abundance of *Coprococcus* [94]. In Hispanic obese children, an evaluation of the microbiota demonstrated more abundance of *B. massiliensisa* compared to normal weight children, who had a higher abundance of *B. plebius* [95].

According to the dietary reference intake (DRI), the recommendation is to consume no more than 35% of fat calories [96]. There is a significant difference in the dairy fat intake among Hispanic populations: Cubans have a higher intake and Dominicans the lowest, while Mexicans and Puerto Ricans have similar fat consumption [97]. The difference in fat intake is more evident when compared to culture across the continent [98], which may contribute to the gut microbiota diversity in different cultures. This is relevant because research on mice identified that a high-fat diet is threatening the gut microbiome composition and causing a lower production of brain-derived neurotrophic factor (BDNF) and cyclic adenosine monophosphate (cAMP). Having low levels of BDNF and cAMP tend to decrease neurotransmitter levels, such as serotonin, a chemical that contributes to well-being and human behavior, which leads to depressive behavior in studied mice [99,100].

Other factors that can also impact the gut microbiome are the consumption of fibers and proteins. Dietary fibers are nourishments, such as vegetables and fruits, which contain carbohydrates. When carbohydrates are digested by microbial enzymes and fermented, SCFAs are produced as previously explained [86]. An example of a SCFA-producing bacteria are the *Bacteroides* (*Bacteroides caccae*, *Bacteroides vulgatus*, *Bacteroides ovatus*, *Bacteroides dorei*, and *Bacteroides uniformis*) [101]. These bacteria are essential to the gut microbiome. Studies identified a negative correlation between levels of *Bacteroides*, quality of life, and depression symptoms [70,102]. Proteins are also vital for *Bacteroides*; people who have animal protein intake in their diet have enriched Bacteroides in their microbiota [86]. The recommended dietary fiber intake is 14 g/1000 kcal [103] or 25 g a day for women and 35 g a day for men [104,105]. In the US, Hispanic populations have a higher dietary fiber intake, but its consumption is still below daily required values [106]. Moreover, protein intake differs by race/ethnicity; among older people, Hispanic Americans had the highest protein intake compared to European Americans and African Americans [107]. In the United States, Hispanics and the Asian population had relatively more protein intake than non-Hispanic Black and non-Hispanic Whites [108]. 

A balanced diet should contain the correct proportions of carbohydrates, protein, probiotics, and others to help keep the gut microbiome healthy and functioning correctly. The use of probiotics can help to balance and increase gut microbiome diversity. Probiotics are microorganisms that contribute positively to the host’s health, mainly by modulating the gut microbiome [109]. Foods such as yogurt, sauerkraut, and kombucha are rich in probiotics, essential for the gut microbiome. In animal models fed with probiotics, they found that the animals had fewer physiological and behavioral changes [110]. The bi-directional communication by the gut–brain axis could explain how the gut microbiota can affect mood disorders. It is clinically adequate to have two doses of 10^9^–10^10^ colony-forming units (CFU) of probiotics in consumer products [111,112]. A Systematic review, conducted in 2016, concluded that probiotics, such as *Lactobacillus* and *Bifidobacteria*, could positively affect patients with anxiety and depression by decreasing the symptoms associated with the condition [113]. It is still unknown which probiotics could be more effective in different populations. However, the recommendation is to include probiotic meals in our diet to provide good composition and diversity in our gut microbiome. Clinical research with patients of anorexia nervosa—an eating disorder in which people are dangerously underweight—showed lower bacterial diversity and depressive and anxious behavior [114].

## 6. Genomics and the Microbiome

The genome is our total genetic material (DNA), and it has a role in gene regulation, phenotype, and in the microbiome. However, there is a bi-directional relationship between the host phenotype and microbiome. External factors, such as the environment, diet, and medication, can also alter it [115].

Some studies have shown that the microbiome composition could influence our genome. To demonstrate this, hatched chickens were inoculated with two different groups of bacteria. The ileum’s genome expression was associated with the intestinal microbial composition. Each experimental group had different gene expressions; however, chickens within the same group had more similar fingerprints than those from the other groups. In addition, the gene’s expressions were compared, and data showed that ion transport genes were most expressed in one of the inoculated groups suggesting that gut microbiota correlates to gene control [116]. 

Heritability is a measure in genetics that collects information on an individual’s genes and traits to analyze at a population scale. It has also been used as a disease risk diagnosis tool to study whether specific genes related to the illness are passed down through generations. These measures are used primarily as a starting point in studies to identify a trait variance and then focus on elements that affect them. Additionally, delivery methods have a role in the “inherited” microbiome. Babies born by cesarean section have a microbiota similar to their mother’s skin, while those born vaginally have an enriched microbiota similar to the mother’s vagina [117]. Differences in microbiota have been linked to weak immune systems and chronic diseases in infants born by cesarean [118]. However, a clinical study has shown that babies delivered by cesarean could receive vaginal microbiota if wiped with gauze inoculated with vaginal fluid from the mother. The microbiota can be partially restored to be similar to those delivered vaginally [119]. 

Studies in this topic have focused on human microbiome heritability in addition to considering diet and environment as influencing gut microbiota composition factors. Evaluation of the gut microbiota composition of monozygotic (MZ) and dizygotic (DZ) adult twins of European ancestry, African ancestry, and Malawian found no significant difference [34,120]. These data imply that microbiome heritability is low. However, a more extensive study of the gut microbiome of twins from the United Kingdom confirmed the microbiome’s significant heritability, including specific bacteria and families *Christensenellaceae*, *methanogenic Archaea*, *genus Tenericutes*, and *Bifidobacteriaceae* [28]. A previous study associated *Christensenellaceae* abundance in twins with lower BMI, raising doubt about whether the microbiome can shape the phenotype. The *Christensenellaceae* family was then tested in inoculated mice to confirm the observations in humans. The inoculated mice presented reduced weight gain when evaluated against control groups. These data suggest the microbiome is an intermediator in the individual’s genome and physical characteristics [121]. On the other hand, it has been demonstrated that genetically unrelated couples had more microbiota similarities than genetically related members of different families. This suggests that people who live together are more alike in their gut composition than those who do not but are genetically related, thereby indicating the environmental effect on gut microbiota over the heritability [122,123,124].

Other studies have evaluated the association between the human gut microbiome and host genetic variations. The presence or absence of *Ruminococcus* and *Coprococcus* was associated with being homozygous or heterozygous for specific genetic variants. Those patients were less likely to have the bacteria than those without the variants [125]. Changes in the abundance of *Bifidobacterium* and Firmicutes are associated with specific gene variants observed in genome-wide association study (GWAS). However, the associations could be affected by cofounders. Some genes are associated with particular diseases, and microbiome changes are linked to the same conditions [125].

Genome-wide scans are an excellent resource for researching the relationship between the microbiome and the genome [126]. A metagenome-wide association study (MGWAS) sought to sequence the gut genome of 345 Chinese subjects (with type 2 diabetes (T2D) and healthy individuals) to identify disease-associated indicators in the gut genome for diagnosis purposes. The T2D patients were found to have only moderate gut microbial dysbiosis, but their composition significantly differed from healthy subjects. Their dysbiosis included low levels of butyrate-producing bacteria, and several pathogens’ levels were higher than in healthy individuals [127]. Moreover, the heritability of the microbiome has been investigated as a possible marker to identify IBD probability in humans via gut microbiota and genome analysis. Genome analysis identified a significantly higher genetic risk of IBD in diagnosed patients versus individuals without a IBD diagnosis. The microbiome analysis showed microbiome dysbiosis (general reduction in microbial diversity) in IBD patients [128]. Genome studies linked to microbial communities have shown that over time, genetic variations and single-nucleotide polymorphisms (SNP) occur in microbial genomes at different rates. Some species, such as *Ruminococcus torques*, *Streptococcus parasanguinis*, and *Faecalibacterium prausnitzii*, have more significant genetic changes compared to other species, such as *Bifidobacterium angulatum*, *Methanobrevibacter smithii*, and *Alistipes putredinis* [129]. Those genetic changes in microbial genomes have been linked to different phenotypes such as depression, changes in BMI, and blood pressure. Deletion of more than a thousand base pairs (structural variant) in the *Collinsella* sp. genome was associated with depression onset [129]. Additionally, microbial genome changes, microbial abundance, and compositional changes were associated with patients with depression.

On the other hand, people with depression and anxiety have gut dysbiosis and increased gut barrier permeability compared to healthy individuals. The permeability was characterized by the increased plasma levels of lipopolysaccharide (LPS), zonulin, and fatty acid-binding protein-2 (FABP2). These findings suggest that the gut can be a treatment target for these psychological disorders in patients with no physical symptoms of a gut disease [130]. Patients with depression are often treated with antidepressants to decrease depressive symptoms and keep them from returning. Selective serotonin reuptake inhibitor (SSRI) antidepressants are the best treatment for most patients with depressive symptoms. It is the first line of treatment recommended in the guidelines, such as the National Institute for Health and Care Excellence in the UK [131] and the American Psychological Association [132]. The way SSRIs work is by maintaining the levels of serotonin stable enough to prevent depressive symptoms.

A study in mice found that antidepressants reduce gut bacterial richness such as *Ruminococcus*, *Adlercreutzia*, and *Alphaproteobacteria*. Furthermore, they observed that mice supplemented with *Ruminococcus flavefaciens* had decreased depressive-like behavior compared with mice supplemented with *Adlercreutzia equolifaciens* [133]. The gut microbiome can affect the SSRI effectiveness in the body [134], and SSRIs can affect the gut’s bacterial composition. Some SSRIs, such as citalopram, alprazolam sertraline, fluoxetine, and paroxetine, can act as antimicrobials in the intestines [135]. Future research expects to find the effect of antidepressants in different racial and ethnic groups’ gut microbiome.

## 7. Hispanics: Why We Should Understand Their Microbiome?

Hispanic and Latinos are the second largest ethnic group in most US states, and among the first in California, New Mexico, and Texas, with nearly the same proportion as Caucasians (39.7% Whites vs. 39.3% Latinos) [55]. Worldwide, Latinos represent 8.42% of the total world population [136], with admixture typically inherited through a mix of European, Native American, and African ancestry. Adding these already unique genomic variations, they represent a diversity of lifestyles, environments, and even dietary patterns. This makes them a complex group to study, added to the fact that they have been found to be at higher risk for chronic diseases [137,138]. Hispanics have higher rates of obesity than non-Hispanic Whites [139], and there are also many disparities among Hispanic subgroup such as a lower birth weight rate for Hispanic infants in comparison to non-Hispanic Whites [140]. The literature has shown that depressive symptoms in Hispanics are associated with financial stress, low education, and chronic medical conditions as well as their religious beliefs [141,142]. 

Hispanics/Latinos from diverse countries express their emotions in variable ways depending on their country of origin. Studies have shown that Hispanics born in the United States tend to have more prevalence of depression than Hispanics born in their country of origin [143], and those without religious beliefs are more prone to anxiety or depression [144]. Hispanic background and origin influence the likelihood of depression symptoms. Individuals with a Puerto Rican background are more likely to present anxiety and depression symptoms than those with Mexican background [145]. In addition, different racial and ethnic groups differ in the amounts of antidepressant consumption. The Hispanic community’s antidepressant use is the lowest compared with other racial groups in the US [143]. A study found that Hispanic Americans and Asian Americans showed high depressive symptoms but low levels of antidepressant use than Non-Hispanics Whites, which could be related to socioeconomical barriers [146]. In the Hispanic community, people with Puerto Rican backgrounds showed the highest depressive prevalence and the highest proportion of antidepressant use [147]. There are so far no studies on the impact of antidepressant use and the gut microbiome in Puerto Ricans and very few on other Hispanic populations [148,149]. 

In addition, there is significant interest in studying Hispanic populations, because results have shown that Hispanic nutrition and health-related psychological factors tend to be worse than in other racial groups such as Non-Hispanics Blacks and Whites [150]. Investigation conducted with different Hispanic communities found that gut microbiome composition depended on migration and sociodemographic status [38]. This evidently is a call to action on research to elucidate the link between lifestyle, behavior, and the gut microbiota’s composition and diversity among Hispanic populations and how these could be linked to anxiety and depression symptoms.

Several programs are currently sequencing the microbiomes of populations from multiple races, but there is a shortage of Latin American people being studied with a slight rise each year [35]. The main reasons why microbiome studies in Latino communities are lacking resides in the fact that there are still many challenges for scientists in low- and middle-income countries (LMICs) to pursue these studies, reflecting the lack of equity in the field. Additionally, there is not enough training for faculty and graduate students due to the language and communication barriers to create inclusive microbiome training programs to engage new generations of scientists to develop them [36]. Data analyses and bioinformatics applied to microbiology are still courses that are not part of most graduate programs and the traditional workshops clearly do not suffice. Studies conducted on specific Hispanic populations could broaden our understanding of the gut–brain axis. Furthermore, the limited research on these ethnicities generates a wide gap of information needed to understand the factors that affect the human microbiome and disease. 

## 8. Conclusions

This review summarizes studies that have established an association between the gut microbiome and anxiety–depressive behavior through the gut–brain axis. The gut microbiota can modulate neurotransmitter production, thus being linked with the severity of depressive symptoms. Environmental factors, diet, nutrient intake, medication, and lifestyle can also alter the microbiota composition and diversity, leading to psychological disorders. Different racial and ethnic groups have a unique gut microbiome composition, because they have different environments and lifestyles. That could be one of the reasons why some populations exhibit more prevalence in psychological disorders and have different responses to treatments. This article shows the importance of studying the gut microbiome and its effect on patients with anxiety and depressive symptoms.

At present, there have not been many studies on the Hispanic population to understand the gut–brain axis. Understanding the relationship between the gut–brain axis in the Hispanic population would help develop advanced treatments for these patients in pharmaceutical studies and expand the knowledge of the human body’s physiology. This work must serve as a call out for funding studies on gut microbiota, pharmacogenetics, depression, and anxiety behavior among Hispanic populations. Low-income countries have a vast geographical diversity of the microbial biosphere, yet they lack funding to pursue this specific research focus.
